# 1-Aminocyclopropane-1-carboxylic acid as a signalling molecule in plants

**DOI:** 10.1093/aobpla/plt017

**Published:** 2013-03-11

**Authors:** Gyeong Mee Yoon, Joseph J. Kieber

**Affiliations:** Department of Biology, University of North Carolina, Chapel Hill, NC 27599-3280, USA

**Keywords:** 1-Aminocyclopropane-1-carboxylic acid, ACC synthase, cell elongation, cell signalling, cell wall, ethylene, LRR-RLKs

## Abstract

Ethylene influences many aspects of plant growth and development including germination, leaf and floral senescence and abscission, fruit ripening, and the response to abiotic and biotic stress. The pathways involved in the biosynthesis of and response to ethylene have been elucidated. The first committed and generally rate-limiting step in ethylene biosynthesis is the conversion of *S*-adenosyl-methionine to 1-aminocyclopropane-1-carboxylic acid (ACC) by ACC synthase (ACS). This enzyme is encoded by a gene family in most plants and is subject to both transcriptional and post-transcriptional control in response to numerous endogenous and environmental cues. In addition to its well-described function as the immediate precursor of ethylene, recent studies suggest an alternative, non-canonical role for ACC. These studies found that in some contexts, chemical inhibitors of ethylene biosynthesis caused effects on root cell expansion that were not observed when ethylene perception or signalling was disrupted, suggesting that ACC, but not ethylene, played a role in these specific processes. Furthermore, mutation of all eight *ACS* genes in *Arabidopsis* was found to result in embryo lethality, in contrast to the relatively modest developmental effects of null mutants in the ethylene signalling components. This divergence suggests that ACC may play a role as a signalling molecule in plants. Here, we first review the ethylene biosynthesis pathway and then discuss these studies suggesting an independent role of ACC as a signalling molecule.

## Abstract (precis)

This review summarizes and discusses the role of ACC synthase in plants. The classic role of ACC synthase is to act as the key enzyme in the biosynthetic pathway for the plant hormone ethylene. Several recent papers have converged on the notion that ACC, the immediate product of ACC synthase, acts as a novel signaling molecule in plants independent of its conversion to ethylene. The evidence for this hypothesis from these papers and potential roles for ACC is summarized and discussed.

## Introduction

**Figure 1.**
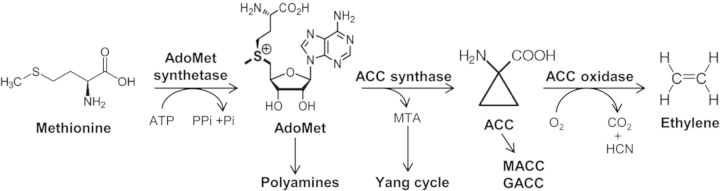
Ethylene biosynthetic pathway. The enzymes catalysing each step are shown above the arrows. In addition to its role in ethylene biosynthesis, AdoMet serves as a precursor for polyamine biosynthesis. The MTA product of ACS is recycled back to methionine via the Yang cycle. AdoMet, *S*-adenosyl-methionine; Met, methionine; ACC, 1-aminocyclopropane-1-carboxylic acid; MTA, methylthioadenine.

The simple gas ethylene has been recognized as a plant hormone for almost a century (Neljubov 1901; Crocker and Knight 1908; Knight *et al.* 1910; Funke *et al.* 1938). It has been shown to influence a diverse array of plant growth and developmental processes, including germination, leaf and flower senescence and abscission, nodulation, fruit ripening, and the response to a wide variety of stresses (Mattoo and Suttle 1991; Abeles *et al.* 1992). Ethylene biosynthesis is tightly regulated. It is elevated during a number of developmental events such as germination, leaf and flower senescence and abscission, and fruit ripening as well as in response to a diverse group of stimuli, including biotic and abiotic stress, wounding, light, and other hormones, such as auxin, cytokinin, brassinosteroid and ethylene itself. The biosynthesis of ethylene begins with the conversion of the amino acid methionine to *S*-adenosyl-methionine (AdoMet) by the enzyme AdoMet synthetase (Fig. 1) (Yang and Hoffman 1984; Kende 1993; Zarembinski and Theologis 1994). *S*-adenosyl-methionine serves as a precursor in a number of biosynthetic pathways, including the production of polyamines. 1-Aminocyclopropane-1-carboxylic acid synthase (ACS) catalyses the conversion of AdoMet to 1-aminocyclopropane-1-carboxylic acid (ACC), which is the first committed and in most instances the rate-limiting step in ethylene biosynthesis. 1-Aminocyclopropane-1-carboxylic acid synthase is a pyridoxal-5′-phosphate-dependent enzyme that is evolutionarily related to the amino transferase superfamily. 1-Aminocyclopropane-1-carboxylic acid is converted to ethylene by ACC oxidase (ACO), a member of the oxygenase/oxidase superfamily of enzymes (Fig. 1). Both ACS and ACO are encoded by multigene families that are differentially regulated by various developmental and environmental inputs (Barry *et al.* 1996; Chen and McManus 2006).

The level of ACS activity closely parallels the level of ethylene production in most plant tissues (Acaster and Kende 1983; Yang and Hoffman 1984; Mattoo and Suttle 1991), although under conditions of high ethylene production, such as fruit ripening, ACO can become rate limiting. A gene encoding ACS was first cloned from zucchini (Sato and Theologis 1989), and it has since been cloned from many other plant species. Analysis of the purified *Arabidopsis* ACS proteins reveals a diversity of kinetic properties (i.e. various affinities for the substrate AdoMet and different *k*_cat_ values) (Yamagami *et al.* 2003). This diversity is further enhanced by heterodimerization among the various ACS isoforms (Tsuchisaka and Theologis 2004). This biochemical diversity suggests that ACS isoforms may be optimized for different roles in various tissues and cell types.

1-Aminocyclopropane-1-carboxylic acid synthase proteins can be divided into three groups based on the sequences of their C-terminal domains, which impart distinct properties governing the stabilities of the proteins (Chae and Kieber 2005; McClellan and Chang 2008). Type-1 ACSes share a highly conserved C-terminal sequence, including both a putative calcium-dependent protein kinase (CDPK) phosphorylation target site and three mitogen-activated protein kinase (MAPK) phosphorylation sites; type-2 ACSes have only the predicted CDPK site; and type-3 ACS proteins lack both sites. The stability of type-1 ACSes is regulated by MAPK-mediated phosphorylation in response to pathogen infection, leading to accumulation of type-1 ACS proteins and therefore enhanced ethylene production (Kim *et al.* 2003; Liu and Zhang 2004; Joo *et al.* 2008). The C-terminal TOE (Target of ETO1) domain of the type-2 ACS proteins interacts with the ETHYLENE-OVERPRODUCER1 (ETO1)/ETO1-LIKE (EOL) proteins, which act as components of E3 ligases to ubiquitinate and hence target these ACS proteins for degradation by the 26S proteasome (Chae *et al.* 2003; Wang *et al.* 2004; Yoshida *et al.* 2005, 2006; Christians *et al.* 2009). The role of phosphorylation in the regulation of type-2 ACS protein stability has not been well established. The C-terminal domain of type-3 ACS proteins is very short and lacks CDPK and MAPK target sites, suggesting that this isoform may be more stable as compared with other ACS proteins. Recently, a ring-type E3 ligase, XBAT32, has been shown to play a role in the regulation of type-3 and type-2 ACS protein stability via targeting to the 26S proteasome pathway (Lyzenga *et al.* 2012).

A further level of control on the production of ethylene is the conjugation of ACC to an inactive form, malonyl-ACC (MACC), by the enzyme ACC malonyltransferase (Amrhein *et al.* 1981; Kionka and Amrhein 1984; Jiao *et al.* 1986). This is similar to other phytohormones that are also rendered inactive via conjugation, although in most cases these other hormones are conjugated to various sugar moieties (Schreiber *et al.* 1986). A second ACC conjugate, 1-(γ-l-glutamylamino) cyclopropane-1-carboxylic acid (GACC), has also been identified from tomato fruits (Martin *et al.* 1995). There is some evidence that the level of ACC malonyltransferase is regulated, which may contribute to controlling ethylene production in various plant tissues (Liu *et al.* 1985; Philosoph-Hadas *et al.* 1985; Jiao *et al.* 1986; Gallardo *et al.* 1991). There is also evidence to suggest that MACC can be hydrolysed to ACC and can thus contribute to the pool of active ACC (Jiao *et al.* 1986).

## The Emerging New Role of ACC in Plant Growth and Development: ACC as a Signal?

While it is clear that a major role of ACC is to act as the precursor of ethylene, several recent studies have converged on the idea that ACC itself acts as a signal independent of its conversion to ethylene. Early studies indicated that ACC is transported from the root to the shoot when plants are stressed, resulting in an increase in ethylene biosynthesis in the shoots. For example, ACC levels are elevated in roots of tomatoes in response to waterlogging or anaerobiosis, and ACC is then transported to the shoot via the xylem, resulting in a stimulation of ethylene production in the shoot (Bradford and Yang 1980; English *et al.* 1995). Similarly, rehydration of drought-stressed *Cleopatra mandarin* plants resulted in root-to-shoot transport of ACC, and subsequent leaf abscission as a result of elevated ethylene biosynthesis (Tudela and Primo-Millo 1992). These results suggest that ACC acts as a signal in root-to-shoot communication, although in these systems it is likely that ethylene, rather than ACC itself, is ultimately the active molecule, at least in the shoots.

Two recent studies have demonstrated that ACC itself may act as a signal in some contexts. These studies found that cell expansion phenotypes in roots can be reversed by inhibitors of ethylene biosynthesis, but not by chemical (e.g. 1-methylcyclopropene (1-MCP) or silver ions) or genetic disruption of ethylene perception or signalling (Xu *et al.* 2008; Tsang *et al.* 2011). This divergence between the effects of inhibition of ethylene biosynthesis and signalling strongly suggests that ACC has a function independent of its role in ethylene biosynthesis and that ACC itself may act as a signal independent of ethylene.

In the first study, the authors examined the role of the FEI1 and FEI2 leucine-rich repeat receptor-like protein kinases (LRR-RLKs) in the growth of roots in *Arabidopsis*. Disruption of both FEI1 and FEI2 resulted in a defect in anisotropic root growth. In wild-type root cells, radially orientated cellulose microfibres in the wall constrict radial expansion, resulting in primarily longitudinal growth. In the *fei1 fei2* mutant, there is significantly less cellulose in the root tip as compared with the wild type, accounting for the loss of growth anisotropy. Growth of the *fei1 fei2* mutant in the presence of α-aminoisobutyric acid (AIB), which is a structural analogue of ACC that blocks ACO activity by acting as a competitive inhibitor of the ACC substrate, completely suppressed the root phenotype of *fei1 fei2* and also reversed the defect in cellulose biosynthesis in *fei1 fei2* (Xu *et al.* 2008). Likewise, aminooxy-acetic acid (AOA), which inhibits enzymes that require pyroxidal phosphate, including ACS, also reversed the *fei1 fei2* swollen root phenotype. As AOA and AIB block ethylene biosynthesis by distinct mechanisms, it is unlikely that this phenotypic reversion of *fei1 fei2* is due to off-target effects. Furthermore, this is not a general effect of AIB as it did not reverse the root swelling phenotype of the *cob* mutant (Xu *et al.* 2008), which also displays swollen roots as a result of defects in cellulose biosynthesis (Schindelman *et al.* 2001). Thus, ACS function is required for the growth defect in *fei1 fei2* mutant roots. In contrast to the inhibition of ACS function, neither 1-MCP nor silver ions, both of which block ethylene perception, had any appreciable effect on the root phenotype of the *fei1 fei2* mutant (Xu *et al.* 2008). Likewise, neither *etr1*, a mutation in an ethylene receptor, nor *ein2*, a strong ethylene-insensitive mutant that acts downstream of ETR1, suppressed the *fei1 fei2* root phenotype (Xu *et al.* 2008). These results suggest that ACS plays a role in the FEI pathway and that swelling in the absence of FEI depends either on a hitherto undiscovered pathway for ethylene perception or on ACC itself acting as a signalling molecule.

The kinase domain of both FEI1 and FEI2 interacted with type-2 ACS proteins, but not with other classes of ACS proteins (Chae and Kieber 2005). This interaction did not require FEI kinase activity, and FEI did not phosphorylate ACS5 protein *in vitro*, consistent with the observation that kinase activity is not essential for FEI function *in vivo*. Measurements of ethylene production revealed that root tissues from wild-type and *fei1 fei2* mutant seedlings made comparable amounts of ethylene (Xu *et al.* 2008). Thus, the FEIs do not appear to affect the overall protein level or catalytic activity of ACS. One model consistent with these results is that the FEIs act as a scaffold to localize a subset of the type-2 ACS proteins to a specific domain of the plasma membrane to generate a localized signal that regulates cellulose synthesis.

A subsequent study also suggested that ACC acts as a signal to regulate cell wall function. Tsang *et al.* (2011) examined the response of root epidermal cells to perturbations of the cell wall. Inhibition of cellulose synthesis via isoxaben treatment inhibited the accelerated elongation phase of root epidermal cells. Surprisingly, this inhibition of cell expansion in response to isoxaben was reversed by inhibitors of ethylene biosynthesis (aminoethoxyvinylglycine (AVG), AOA and 2-anilino-7-(4-methoxyphenyl)-7,8-dihydro-5(*6H*)-quinazolinone), but inhibition of ethylene perception, either chemically or genetically, had no significant effect. Consistent with ACC acting as a signal, treatment with ACC reduced root epidermal cell elongation with kinetics similar to that of isoxaben treatment, and this effect was also observed in the ethylene-insensitive *ein3/eil1* mutant. Similar effects were found when the cell wall was perturbed by treatment with the cell wall-binding dye Congo red, or by treatment with the elicitor flg22. Interestingly, the authors found that isoxaben-induced reduced cell elongation was suppressed by PEO-IAA, a synthetic antagonist of TIR1 receptor function, indicating that auxin signalling is necessary for ACC signalling following perturbation of cell wall function. Likewise, they found that superoxide production is also probably involved in this pathway downstream of ACC. The authors hypothesized that ACC acts through reactive oxygen species and auxin to sense cell wall integrity and, in turn, regulate cell wall synthesis (Tsang *et al.* 2011).

While both these studies indicate a role for ACC in the cell wall sensing system, there are some differences. First, the *fei1 fei2* mutant is not affected in the rapid response to isoxaben in root epidermal cells (Tsang *et al.* 2011), suggesting that these RLKs are not involved in this particular response. Secondly, the Tsang *et al.* study suggests that ACC acts downstream of perturbation of the cell wall to regulate cell expansion. In the Xu and Kieber study (Xu *et al.* 2008), the FEI/ACC pathway acts upstream to regulate cellulose synthesis. These differences could be explained if the FEIs act to perceive perturbations in the cell wall, and then act in a feedback signalling pathway to increase cellulose synthesis. This feedback could involve ACC, which thus creates a signalling loop between cell wall integrity and synthesis.

An additional study that supports a non-canonical signalling role for ACC is the analysis of knockout lines from the Theologis group (Tsuchisaka *et al.* 2009). This group analysed single and multiple mutants of the eight *ACS* genes in *Arabidopsis* using a combination of T-DNA insertion alleles and amiRNA technology. An amiRNA designed to silence the *ACS8* and *ACS11* genes was introduced into an *acs2*,*4*,*5*,*6*,*7*,*9* hextuple mutant to create a line in which all eight functional *ACS* genes (an octuple mutant) were eliminated or reduced in function. In lines in which the amiRNA transgene modestly down-regulated *ACS8* and *ACS11*, the octuple *acs* mutant displayed a variety of phenotypes, including delayed growth, reduced branching, delayed leaf senescence, smaller cotyledons, altered leaf shape, reduced response to gravity, compromised pathogen response and a loss of the apical hook in etiolated seedlings. Some of these phenotypes resemble those observed in ethylene-insensitive mutants, but others, such as the reduced branching, are not found in ethylene-insensitive mutants. It is important to note that the lines the authors were able to grow to adult plants were not null mutants for the *ACS8* and *ACS11* genes. They also examined octuple *acs* lines that displayed a stronger reduction in *ACS8/ACS11* transcript levels, which were only able to be propagated as heterozygotes as the more severe disruption of the *ACS* genes resulted in embryo lethality. The authors were able to complement the octuple *acs* phenotype by introducing transgenes expressing *ACS8* and *ACS11* cDNAs that had altered amiRNA target sites, indicating that the effects of the amiRNA were specific to these genes. The embryo-lethal phenotype of the strong octuple lines is distinct from the relatively modest effects of mutants that eliminate ethylene perception (Tsang *et al*. 2011). This divergence of the effects of disruption of ethylene biosynthesis and signalling strongly supports the notion that ACS, and thus probably ACC, has a role distinct from its function in ethylene production. Further characterization of these lines should reveal the roles of ACC in plant growth and development that are distinct from its function in ethylene biosynthesis. As with the previous two studies discussed above, the genetic analysis of ACS mutant lines suggests a model in which ACC or some derivative of ACC may act as a signalling molecule. An alternative possibility is that disruption of ACS causes an increase in ACS precursors, most notably AdoMet, which might lead to increased synthesis of other physiologically active molecules, such as polyamines (Harpaz-Saad *et al.* 2012), which may contribute to the developmental effects observed. Polyamines and ethylene have opposing effects on several plant processes, such as senescence and ripening, and these signals have effects on the synthesis of each other (Harpaz-Saad *et al.* 2012).

## Conclusions

A series of diverse studies have converged on a model in which ACC, in addition to its major role as a precursor of ethylene, acts independently as a signal to regulate plant growth and development, most clearly in regulating cell wall function in the root, but also in an essential role for embryo development. Much more work is needed to further define the roles of ACC and the downstream elements mediating its perception and signal transduction. One important question is whether ACC itself or some ACC derivative is the active signalling molecule. Ethylene is not the only metabolic fate of ACC because, as noted above, ACC can be conjugated to a malonyl or a glutamyl group (Amrhein *et al.* 1981; Kionka and Amrhein 1984; Jiao *et al.* 1986; Martin *et al.* 1995). It is possible that a metabolic product of ACC such as these or a distinct metabolite acts as the signal in the FEI pathway, rather than ACC itself. Further, ACS shares AdoMet as a biosynthetic precursor with polyamines and disruption of ACSes may cause an increase in AdoMet, potentially altering polyamine levels, which may also influence growth and development. Measurements of polyamine levels in the ACS knockout lines would be a first step in addressing this possibility. Another important question is what is the basis for the lethality of the multiple ACS knockout: at what stage do the mutant embryos first deviate from the wild type? What developmental processes are disrupted? Are other hormone pathways affected? Another major question to be addressed is how ACC, or its derivative, is perceived in the cell and how this then signals to downstream elements. The answer to these and additional questions will provide unique insight into our understanding of plant growth and development.

## Sources of Funding

Research on ethylene in our laboratory is supported by a grant from the US National Science Foundation.

## Contributions by the Authors

Both authors contributed to a similar extent overall.

## Conflict of Interest Statement

None declared.

## References

[PLT017C1] Abeles FB, Morgan PW, Saltveit ME (1992). Ethylene in plant biology.

[PLT017C2] Acaster MA, Kende H (1983). Properties and partial purification of 1-aminocyclopropane-1-carboxylate synthase. Plant Physiology.

[PLT017C3] Amrhein N, Schneebeck D, Skorupka H, Tophof S, Stockigt J (1981). Identification of a major metabolite of the ethylene precursor 1-aminocyclopropane-1-carboxylic acid in higher plants. Naturwissenschaften.

[PLT017C4] Barry CS, Blume B, Bouzayen M, Cooper W, Hamilton AJ, Grierson D (1996). Differential expression of the 1-aminocyclopropane-1-carboxylate oxidase gene family of tomato. The Plant Journal.

[PLT017C5] Bradford KJ, Yang SF (1980). Xylem transport of 1-aminocyclopropane-1-carboxylic acid, an ethylene precursor, in waterlogged tomato plants. Plant Physiology.

[PLT017C6] Chae HS, Kieber JJ (2005). Eto Brute? The role of ACS turnover in regulating ethylene biosynthesis. Trends in Plant Sciences.

[PLT017C8] Chae HS, Faure F, Kieber JJ (2003). The *eto1*, *eto2* and *eto3* mutations and cytokinin treatment elevate ethylene biosynthesis in *Arabidopsis* by increasing the stability of the ACS5 protein. The Plant Cell.

[PLT017C7] Chen B, McManus M (2006). Expression of 1-aminocyclopropane-1-carboxylate (ACC) oxidase genes during the development of vegetative tissues in white clover (*Trifolium repens* L.) is regulated by ontological cues. Plant Molecular Biology.

[PLT017C9] Christians MJ, Gingerich DJ, Hansen M, Binder BM, Kieber JJ, Vierstra RD (2009). The BTB ubiquitin ligases ETO1, EOL1 and EOL2 act collectively to regulate ethylene biosynthesis in *Arabidopsis* by controlling type-2 ACC synthase levels. The Plant Journal.

[PLT017C10] Crocker W, Knight LI (1908). Effect of illuminating gas and ethylene upon flowering carnation. Botanical Gazette.

[PLT017C11] English PJ, Lycrtt GW, Roberts JA, Jackson MB (1995). Increased 1-aminocyclopropane-1-carboxylate oxidase activity in shoots of flooded tomato plants raises ethylene production to physiologically active levels. Plant Physiology.

[PLT017C12] Funke GL, DeCoeyer F, DeDecker A, Maton J (1938). The influence of the emanation of apples on several life phenomenon of plants. Biologisch Jaarboek.

[PLT017C13] Gallardo M, Delgardo MD, Sanchez-Calle IM, Matilla AJ (1991). Ethylene production and 1-aminocyclopropane-1-carboxylic acid conjugation in thermoinhibited *Cicer arietinum* L. seeds. Plant Physiology.

[PLT017C14] Harpaz-Saad S, Yoon GM, Mattoo AK, Kieber JJ (2012). The formation of ACC and competition between polyamines and ethylene for SAM. Annual Plant Reviews.

[PLT017C15] Jiao X-Z, Philosoph-Hadas S, Su L-Y, Yang SF (1986). The conversion of 1-(malonylamino) cyclopropane-1-carboxylic acid to 1-aminocyclopropane-1-carboxylic acid in plant tissues. Plant Physiology.

[PLT017C16] Joo S, Liu Y, Lueth A, Zhang S (2008). MAPK phosphorylation-induced stabilization of ACS6 protein is mediated by the non-catalytic C-terminal domain, which also contains the *cis*-determinant for rapid degradation by the 26S proteasome pathway. The Plant Journal.

[PLT017C17] Kende H (1993). Ethylene biosynthesis. Annual Reviews of Plant Physiology and Plant Molecular Biology.

[PLT017C18] Kim CY, Liu Y, Thorne ET, Yang H, Fukushige H, Gassmann W, Hildebrand D, Sharp RE, Zhang S (2003). Activation of a stress-responsive mitogen-activated protein kinase cascade induces the biosynthesis of ethylene in plants. Plant Cell.

[PLT017C19] Kionka C, Amrhein N (1984). The enzymatic malonylation of 1-aminocyclopropane-1-carboxylic acid in homogenates of mung bean hypocotyls. Planta.

[PLT017C20] Knight LI, Rose RC, Crocker W (1910). Effects of various gases and vapors upon etiolated seedlings of the sweet pea. Science.

[PLT017C21] Liu Y, Zhang S (2004). Phosphorylation of ACC synthase by MPK6, a stress-responsive MAPK, induces ethylene biosynthesis in *Arabidopsis*. Plant Cell.

[PLT017C22] Liu Y, Hoffman NE, Yang SF (1985). Ethylene-promoted malonylation of 1-aminocyclopropane-1-carboxylic acid participates in autoinhibition of ethylene synthesis in grapefruit flavedo disks. Planta.

[PLT017C23] Lyzenga WJ, Booth JK, Stone SL (2012). The *Arabidopsis* RING-type E3 ligase XBAT32 mediates the proteasomal degradation of the ethylene biosynthetic enzyme, 1-aminocyclopropane-1-carboxylate synthase 7. The Plant Journal.

[PLT017C24] Martin MN, Cohen JD, Saftner RA (1995). A new 1-aminocyclopropane-1-carboxylic acid-conjugating activity in tomato fruit. Plant Physiology.

[PLT017C25] Mattoo AK, Suttle JC (1991). The plant hormone ethylene.

[PLT017C26] McClellan CA, Chang C (2008). The role of protein turnover in ethylene biosynthesis and response. Plant Sciences.

[PLT017C27] Neljubov D (1901). Uber die horizontale Nutation der Stengel von *Pisum sativum* und einiger Anderer. Pflanzen Beitrage und Botanik Zentralblatt.

[PLT017C28] Philosoph-Hadas S, Meir S, Aharoni N (1985). Autoinhibition of ethylene production in tobacco leaf disks: enhancement of 1-aminocyclopropane-1-carboxylic acid conjugation. Plant Physiology.

[PLT017C29] Sato T, Theologis A (1989). Cloning the mRNA encoding 1-aminocyclopropane-1-carboxylate synthase, the key enzyme for ethylene biosynthesis in plants. Proceedings of the National Academy of Sciences of the USA.

[PLT017C30] Schindelman G, Morikami A, Jung J, Baskin TI, Carpita NC, Derbyshire P, McCann MC, Benfey PN (2001). COBRA encodes a putative GPI-anchored protein, which is polarly localized and necessary for oriented cell expansion in *Arabidopsis*. Genes and Development.

[PLT017C31] Schreiber K, Schütte H, Sembnder G (1986). Conjugated plant hormones; structure, metabolism, and function. Proceedings of the international symposium on conjugated plant hormones structure, metabolism and function.

[PLT017C32] Tsang DL, Edmond C, Harrington JL, Nühse TS (2011). Cell wall integrity controls root elongation via a general 1-aminocyclopropane-1-carboxylic acid-dependent, ethylene-independent pathway. Plant Physiology.

[PLT017C33] Tsuchisaka A, Theologis A (2004). Heterodimeric interactions among the 1-amino-cyclopropane-1-carboxylate synthase polypeptides encoded by the *Arabidopsis* gene family. Proceedings of the National Academy of Sciences of the USA.

[PLT017C34] Tsuchisaka A, Yu G, Jin H, Alonso JM, Ecker JR, Zhang X, Gao S, Theologis A (2009). A combinatorial interplay among the 1-aminocyclopropane-1-carboxylate isoforms regulates ethylene biosynthesis in *Arabidopsis thaliana*. Genetics.

[PLT017C35] Tudela D, Primo-Millo E (1992). 1-Aminocyclopropane-1-carboxylic acid transported from roots to shoots promotes leaf abscission in *Cleopatra mandarin* (*Citrus reshni* Hort. ex Tan.) seedlings rehydrated after water stress. Plant Physiology.

[PLT017C36] Wang KL-C, Yoshida H, Lurin C, Ecker JR (2004). Regulation of ethylene gas biosynthesis by the *Arabidopsis* ETO1 protein. Nature.

[PLT017C37] Xu SL, Rahman A, Baskin TI, Kieber JJ (2008). Two leucine-rich repeat receptor kinases mediate signaling linking cell wall biosynthesis and ACC synthase in *Arabidopsis*. Plant Cell.

[PLT017C38] Yamagami T, Tsuchisaka A, Yamada K, Haddon WF, Harden LA, Theologis A (2003). Biochemical diversity among the 1-amino-cyclopropane-1-carboxylate synthase isozymes encoded by the *Arabidopsis* gene family. Journal of Biological Chemistry.

[PLT017C39] Yang SF, Hoffman NE (1984). Ethylene biosynthesis and its regulation in higher plants. Annual Review of Plant Physiology.

[PLT017C40] Yoshida H, Nagata M, Saito K, Wang KL, Ecker JR (2005). *Arabidopsis* ETO1 specifically interacts with and negatively regulates type 2 1-aminocyclopropane-1-carboxylate synthases. BMC Plant Biology.

[PLT017C41] Yoshida H, Wang K, Chang C-M, Mori K, Uchida E, Ecker J (2006). The ACC synthase TOE sequence is required for interaction with ETO1 family proteins and destabilization of target proteins. Plant Molecular Biology.

[PLT017C42] Zarembinski TI, Theologis A (1994). Ethylene biosynthesis and action: a case of conservation. Plant Molecular Biology.

